# Association between Patient- and Partner-Reported Sleepiness Using the Epworth Sleepiness Scale in Patients with Obstructive Sleep Apnoea

**DOI:** 10.3390/life12101523

**Published:** 2022-09-29

**Authors:** Konstantinos Chaidas, Kallirroi Lamprou, John R. Stradling, Annabel H. Nickol

**Affiliations:** 1 Ear, Nose, and Throat Department, Oxford University Hospitals NHS Foundation Trust, Oxford OX39DU, UK; 2 Oxford Centre for Respiratory Medicine, Oxford University Hospitals NHS Foundation Trust, Oxford OX37LE, UK

**Keywords:** sleepiness, excessive somnolence, Epworth Sleepiness Scale, patient reported Epworth score, partner reported Epworth score, obstructive sleep apnea, continuous positive airway pressure, oxygen desaturation index, bed partner

## Abstract

Excessive daytime sleepiness in obstructive sleep apnoea (OSA) is often measured differently by patients and their partners. This study investigated the association between patient- and partner-completed Epworth Sleepiness Scale (ESS) scores and a potential correlation with OSA severity. One hundred two participants, 51 patients and 51 partners, completed the ESS before and three months after initiating CPAP treatment. There was no significant difference when comparing patients’ and partners’ ESS scores at baseline (10.75 ± 5.29 vs. 11.47 ± 4.96, respectively) and at follow-up (6.04 ± 4.49 vs. 6.41 ± 4.60, respectively). There was a strong correlation between patients’ and partners’ ESS scores on both (baseline and follow-up) assessments (*p* < 0.001). There was significant improvement in patients’ and partners’ ESS scores after CPAP therapy (*p* < 0.001). There was no significant difference in patients’ or partners’ ESS scores between patients with mild, moderate or severe OSA. There was no significant correlation between oxygen desaturation index (ODI) and ESS score reported either by patient or by partner. In conclusion, our study revealed a strong correlation between patient- and partner-reported ESS scores. However, neither patient- nor partner-completed ESS scores were associated with OSA severity.

## 1. Introduction

Obstructive sleep apnoea (OSA) is an increasingly prevalent sleep disorder affecting 3–7% of men and 2–5% of women [1]. It is characterized by recurrent episodes of partial or complete upper airway collapse during sleep leading to intermittent airflow limitation, sleep fragmentation, arterial oxygen desaturations, and poor sleep quality. The sleep fragmentation and hypoxemia have a variety of medical and functional consequences including cardiovascular and cerebrovascular disease, glucose intolerance, reduced quality of life and daytime sleepiness [2,3]. Excessive daytime sleepiness is considered as a main symptom in OSA and is experienced by most patients [4], but many of them may deny or minimize its degree.

The multiple sleep latency test and the maintenance of wakefulness test are both objective tools to quantify sleepiness, but are time-consuming, laborious and expensive [5]. In order to measure daytime somnolence in common clinical practice, several questionnaires have been developed. One of the simplest and frequently used is the Epworth Sleepiness Scale (ESS) which was first introduced in 1991 and is a tool used to measure the general level of sleepiness in patients with OSA and other sleep disorders in everyday situations [6].

Although ESS has been widely used in both clinical and research settings, there is still controversy in the literature regarding its value as a screening tool for patients with suspected OSA [7,8,9]. It has been observed that some patients lack insight into the degree of their sleepiness resulting in underestimation of its level. For that reason, many studies attempted to determine if the use of partner-reported ESS score is superior compared to patient- reported ESS score in the evaluation of patients with suspected OSA reporting controversial outcomes [10,11,12]. Therefore, there is still much confusion regarding the utility of partner-completed ESS in identifying OSA and predicting its severity.

The aim of our study was to assess the association between patient- and partner-completed ESS scores and to investigate a potential correlation between ESS and OSA severity as well as the impact of continuous positive airway pressure (CPAP) therapy on ESS score.

## 2. Materials and Methods

### 2.1. Study Protocol

Patients aged over 18 years old accompanied by their partners who were seen at the Oxford Adult Sleep and Ventilation Service Clinic between February 2019 and February 2020, were offered to participate in this prospective study. The study protocol was approved by the Institutional Review Board of Oxford University Hospitals NHS Foundation Trust (Datix ID: 6120).

Participants were excluded from the study if they had previous treatment with CPAP for OSA or prior diagnosis or suspicion of other sleep disorder, if they were unable to complete the ESS or if they had no suitable partner in clinic with them. As “partner” was considered a boyfriend/girlfriend, a spouse or a close relative sharing the same house. Patients without evidence of OSA on the baseline sleep study were also excluded.

**Figure 1:**
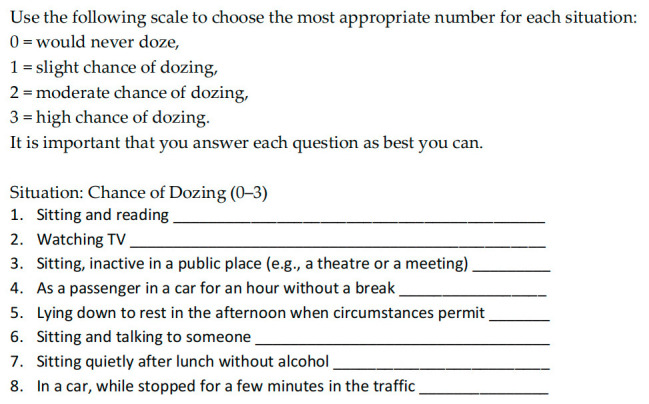
Epworth Sleepiness Scale.

After obtaining verbal consent, patients and their partners completed the ESS independently during the initial visit at the clinic. The ESS is a self-administered questionnaire with 8 questions (Figure 1) [6]. Respondents were asked to rate, on a 4-point scale (0–3), their usual chance of dozing off or falling asleep while engaged in eight different activities. The total ESS score (the sum of 8 item scores) can range from 0 to 24. It has been suggested that a cutoff total score of 10 or higher indicates the presence of hypersomnolence.

The following parameters were also obtained from all patients during their first visit in clinic: age, gender, body mass index (BMI), symptoms correlated to OSA, co-morbidities and current medical treatment, smoking history, alcohol consumption, Mallampati grade and neck circumference. After the baseline screening, patients were scheduled to undergo a diagnostic sleep study with a portable machine. The severity of OSA was evaluated based on the oxygen desaturation index (ODI) and the patients were divided into three groups: mild (5 ≤ ODI < 15 episodes/h), moderate (15 ≤ ODI < 30 episodes/h), and severe (ODI ≥ 30 episodes/h) OSA group. CPAP treatment was offered to all patients that had a diagnosis of OSA. Three months after initiating CPAP therapy both patient and partner were again asked to complete the ESS questionnaire independently.

### 2.2. Statistical Analysis

Data are presented as mean ± standard deviation, as median (minimum–maximum) or as percentage. The data were tested for normality by using the Kolmogorov–Smirnov test. Changes in outcome variables before and after CPAP therapy were compared by Wilcoxon signed-rank test. Kendall rank correlation test was used to assess the relationship between patient- and partner-reported sleepiness, as well as their association with ODI. Kruskal–Wallis test was used for the comparison between mild, moderate, and severe OSA groups and ESS results. The Bland and Altman method was used to plot the difference between partners’ and patients’ ESS scores and measure agreement between them. To investigate the presence of a potential trend, linear regression analysis was performed. *p* values < 0.05 were considered statistically significant. All data were statistically analysed using SPSS software for Windows version 17.0 (SPSS Inc., Chicago, IL, USA).

## 3. Results

A total of 102 individuals (51 patients and 51 partners) participated in the study and completed the ESS before and three months after initiating CPAP treatment. Table 1 shows baseline characteristics, sleep study results and CPAP usage information for all patients. ESS results obtained from all participants and comparisons between patients’ and partners’ total and each item’s scores are presented in Table 2.

Thirty-seven male and 14 female patients participated in the study. There were no gender-related differences between patient- and partner-completed ESS scores (*p* > 0.05).

### 3.1. Baseline ESS

A comparison between patient- and partner-reported total ESS score revealed no significant difference (10.75 ± 5.29 vs. 11.47 ± 4.96, respectively, *p* = 0.157). A comparison of the score for each ESS question separately, revealed no significant difference (*p* > 0.05) except for question 7 (*p* = 0.013). Kendall rank correlation revealed significant correlation between patient- and partner-completed ESS baseline scores (*p* < 0.001, Tb = 0.601).

**Figure 2:**
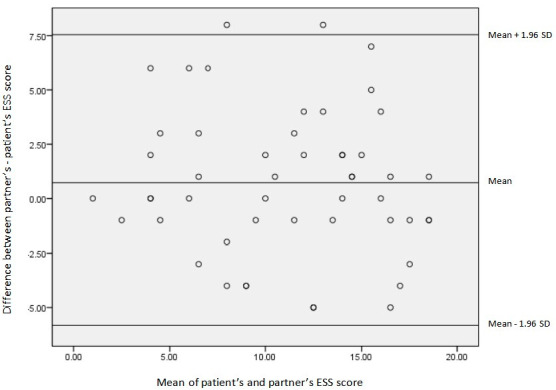
The ESS for paired comparisons is displayed using a Bland–Altman plot. *Y*-axis shows the difference in ESS score (partner’s score minus patient’s score). *X*-axis shows the mean ESS score (mean score of patient- and partner-reported ESS score). Each dot refers to one patient. The midline indicates the mean difference between the two measurements (partners’ minus patients’ ESS score). The upper and lower lines indicate the 95% confidence limits for these measures (upper line: mean difference plus 1.96 × SD, lower line: mean difference minus 1.96 × SD, SD: standard deviation).

The Bland–Altman plot (Figure 2) shows the individual differences between the two ESS measurements for each patient against the mean ESS score. It did not demonstrate statistically significant differences between partners’ and patients’ ESS scores (*p* = 0.467, R^2^ = 0.011).

### 3.2. Follow-Up ESS

A comparison between patients’ and partners’ total ESS scores revealed no significant difference (6.04 ± 4.49 vs. 6.41 ± 4.60, respectively, *p* = 0.128). An additional comparison of the score for each ESS question separately revealed no significant difference (*p* > 0.05) for all the questions. Kendall rank correlation test revealed significant correlation between patient- and partner-reported ESS follow-up scores (*p* < 0.001, Tb = 0.651).

### 3.3. Patients’ ESS before and after Treatment

There was statistically significant improvement (reduction) in patient- reported ESS score post CPAP therapy (10.75 ± 5.29 vs. 6.04 ± 4.49, *p* < 0.001). There was a significant reduction in the score for each ESS question (*p* < 0.05) except for question 8 (*p* = 0.132).

### 3.4. Partners’ ESS before and after Treatment

There was statistically significant improvement in partners’ ESS score after CPAP treatment (11.47 ± 4.96 vs. 6.41 ± 4.60, *p* < 0.001). There was a significant reduction in the score for each ESS question (*p* < 0.05) except for questions 6 (*p* = 0.134) and 8 (*p* = 0.084).

### 3.5. Baseline ESS and OSA Severity

Patients were divided into three groups based on OSA severity as determined by ODI (mild, moderate or severe OSA) and Table 3 shows patients’ and partners’ ESS scores in these groups. There was no significant difference between the groups when comparing the ESS score reported either by patients (*p* = 0.534) or by partners (*p* = 0.858). Likewise, a comparison of patients’ ESS score vs. partners’ ESS score within the same OSA group revealed no significant difference as shown in Table 3.

Kendall rank correlation test showed no significant correlation between ODI and baseline ESS score reported either by patient (*p* = 0.993, Tb = 0.001) or by partner (*p* = 0.794, Tb = −0.026).

## 4. Discussion

Our study investigated the association between patient- and partner-reported ESS score demonstrating that a strong correlation exists. Patients and their partners agree in the perception of patient’s sleepiness suggesting that the value of ESS in assessing patients for suspected OSA is similar regardless the person completing the questionnaire (patient or partner). On the other hand, it seems that neither patient- nor partner-reported ESS scores are associated with OSA severity and, thus, OSA severity cannot be predicted by ESS alone.

The ESS as a subjective tool to quantify somnolence may be inaccurate in certain situations, especially if the patient is not fully aware of the problem or the partner gives an inaccurately positive estimate of patient’s sleepiness. Kumru et al. [10] found a discrepancy between patient’s and partner’s perception of patient’s sleepiness with patients rating their sleepiness lower than their partners. Another study reported that ESS scores of bed partners were higher than those of patients in 67% of the cases suggesting that either the patients tend to underestimate the degree of their sleepiness or their partners overestimate it [12].

In contrast, we demonstrated that partner-completed ESS scores were similar to ESS scores reported by patients. This was also evident by using the Bland–Altman method showing no significant bias between patients’ and partners’ ESS outcomes. An additional comparison of each ESS item scores revealed no significant difference between patient and partner except for question 7 (“sitting quietly after lunch without alcohol”). Interestingly, this is also the question with the greatest disagreement between patient and partner in the study by Kumru et al. [10]. Although the combination of patient- and partner-completed ESS may help the clinician and increase the accuracy of the results, according to our study findings, the addition of ESS score obtained by the partner does not add much value in the screening process for OSA.

Previous studies have investigated the utility of patient- and partner-completed ESS in predicting OSA severity with contradictory results. Several reports suggest that patients’ ESS score does not correlate with OSA severity [8,11,13,14,15,16], whereas other studies show that an association is present [12,17,18,19,20,21]. A few studies have also indicated an association between partners’ ESS score and OSA severity [12,17,22,23].

A study by Bhat et al. [11] revealed a correlation between partner-completed ESS score and apnoea-hypopnoea index (AHI), but not with ODI or other related parameters. Moreover, the authors found that neither patient- nor partner-completed ESS score alone can predict the severity of OSA. Another study reported that both patients’ and partners’ ESS scores were independent of AHI levels and, thus, ESS was found to be a poor predictor of AHI [22]. Similarly, Barry et al. [24] demonstrated that the ESS does not correlate with the AHI. In contrast, Walter et al. [12] documented a significant correlation between ESS as estimated by either the patient or the partner and OSA severity. Nevertheless, the authors were unable to identify a cut-off ESS score that correlated strongly with the presence of severe OSA and suggested that their findings may be partially due to the fact that ESS is a subjective questionnaire which can be influenced by a variety of factors such as the quality and duration of sleep the previous night and personality traits.

In light of these contradictory findings, our findings show no correlation between patient- as well as partner-completed ESS and ODI. Therefore, neither patients’ nor partners’ ESS score can predict OSA severity.

Excessive daytime sleepiness is a common symptom in OSA. Several studies have demonstrated that daytime somnolence levels, both subjective and objective, are not reflective of the severity or even of the presence of OSA [7,8,9,19,25,26,27]. This may explain why ESS score may be misleading when used to evaluate patients for OSA. Several factors other than the severity of OSA as demonstrated by sleep study parameters seem to have a significant impact on the degree of daytime sleepiness.

CPAP has been established as the most effective treatment of OSA and has been shown to be associated with a significant reduction in daytime sleepiness in these patients, as well [28,29]. However, on some occasions, sleepiness may persist despite CPAP therapy. In a previous study, 40% of patients with moderate-severe OSA reported sleepiness after three months of treatment with CPAP [30]. Bonsignore et al. showed a prevalence of persistent daytime sleepiness in CPAP-treated patients around 40% in the first three months and 10–20% after that [31]. The same study showed that patients with daily sleepiness at follow-up were younger and more obese, had slightly more severe OSA and were sleepier at baseline. On the other hand, Patel et al. [32] revealed that CPAP therapy significantly reduced the ESS score by a mean of 2.9 points as compared with placebo. On similar lines, another study showed that patients with moderate and severe OSA had a significant improvement in ESS score after one and three months of CPAP therapy [33].

Our results support that patient-completed ESS score, regardless OSA severity, not only significantly improves after 3 months of CPAP therapy, but also normalizes. There was statistically significant reduction in the score for each ESS question except for question 8, which could be explained by the fact that this item is considered as a low soporific situation and, thus, the patients had a low baseline score.

To our knowledge, no other studies have looked at partner-completed ESS after CPAP treatment to assess post therapy patients’ daytime sleepiness. We found that there is a statistically significant improvement in partners’ ESS score after 3 months of CPAP treatment with a significant reduction in the score for each ESS item except for questions 6 and 8, which are low soporific items. We also found that there was no discrepancy between patient- and partner-completed ESS scores after CPAP treatment.

This study has certain limitations. First, due to the number of participants, conclusions should be made with caution. The patients did not undergo full polysomnography for the diagnosis of OSA. Instead, all patients had a type-3 home sleep study using two respiratory variables, one cardiac variable, one arterial oxygen saturation. Therefore, there were no available data regarding sleep stages, sleep efficiency, number of arousals during sleep, total sleep time, and the presence or not of periodic limb movement (PLM) during sleep. Finally, we used ODI instead of the AHI to make the diagnosis of OSA and classify its severity. However, although AHI is widely used, ODI is as valuable as AHI in diagnosing and grading OSA [34].

## 5. Conclusions

In contrast with previous studies showing a discrepancy between patients’ and partners’ ESS scores, our study revealed an association between patient- and partner-reported sleepiness as measured by ESS. In addition, we found that there was no association between ESS score as estimated by either patient or partner and ODI, and hence, OSA severity cannot be predicted by using ESS. Considering the contradictory data in the literature and the growing recognition of daytime sleepiness and its impact on patients’ quality of life, more studies are required to investigate the utility of patient- and partner-completed ESS. The ultimate aim should be the development of an accessible, convenient and accurate measure of sleepiness, especially in patients with OSA.

## Figures and Tables

**Table 1:** Patients’ baseline characteristics, sleep study results and CPAP usage information (*n* = 51).

Variables	Results
Male sex	37 (72.5)
Age, years	60.21 ± 12.67
Smoking	
Non-smokers	40 (78.4)
Ex-smokers	4 (7.8)
Smokers	7 (13.7)
Comorbidities	
Hypertension	26 (51.0)
Depression	8 (15.7)
Gastroesophageal reflux	7 (13.7)
Diabetes	5 (9.8)
Asthma	4 (7.8)
COPD	3 (5.9)
None	14 (27.5)
BMI, kg/m^2^	35.07 ± 6.23
Neck circumference, cm	42.91 ± 3.64
Mallampati scale	
Grade 1	11 (21.6)
Grade 2	28 (54.9)
Grade 3	10 (19.6)
Grade 4	2 (3.9)
ODI, episodes/hour	35.91 ± 23.37
CPAP use, hours/day	4.89 ± 2.46

Values are given as mean ± standard deviation or as number (%). CPAP: continuous positive airway pressure, COPD: Chronic Obstructive Pulmonary Disease, BMI: body mass index, ODI: oxygen desaturation index.

**Table 2:** Total and each question’s ESS scores prior to (baseline) and three months after (follow-up) initiating CPAP therapy.

ESS Score	Patient-Reported		Partner-Reported		ESS Difference (Partner’s Minus Patient’s Score)	*p* Value
Baseline	Mean	Median	Mean	Median	Mean	
Total	10.75 ± 5.29	11 (1–19)	11.47 ± 4.96	12 (1–19)	0.72 ± 3.341	0.157
Q1	1.84 ± 1.065	2 (0–3)	1.92 ± 1.036	2 (0–3)	0.08 ± 0.796	0.537
Q2	2.02 ± 0.927	2 (0–3)	2.10 ± 0.922	2 (0–3)	0.08 ± 0.796	0.512
Q3	0.88 ± 0.791	1 (0–3)	0.78 ± 0.808	1 (0–3)	−0.10 ± 0.755	0.354
Q4	1.49 ± 1.102	2 (0–3)	1.71 ± 1.119	2 (0–3)	0.22 ± 1.026	0.131
Q5	2.24 ± 1.012	3 (0–3)	2.35 ± 1.016	3 (0–3)	0.11 ± 1.013	0.335
Q6	0.43 ± 0.608	0 (0–2)	0.43 ± 0.700	0 (0–2)	0.00 ± 0.721	0.980
Q7	1.61 ± 1.133	2 (0–3)	1.94 ± 1.047	2 (0–3)	0.33 ± 0.931	0.013 *
Q8	0.25 ± 0.483	0 (0–2)	0.25 ± 0.523	0 (0–2)	0.00 ± 0.529	1.000
Follow-up						
Total	6.04 ± 4.49	5 (0–19)	6.41 ± 4.60	6 (0–19)	0.37 ± 2.537	0.128
Q1	0.88 ± 0.816	1 (0–3)	1.02 ± 0.905	1 (0–3)	0.14 ± 0.633	0.127
Q2	1.27 ± 0.827	1 (0–3)	1.25 ± 0.891	1 (0–3)	−0.02 ± 0.648	0.827
Q3	0.53 ± 0.758	0 (0–3)	0.49 ± 0.674	0 (0–2)	−0.04 ± 0.631	0.655
Q4	0.65 ± 0.868	0 (0–3)	0.84 ± 0.967	1 (0–3)	0.19 ± 0.917	0.108
Q5	1.37 ± 1.076	1 (0–3)	1.39 ± 1.115	1 (0–3)	0.02 ± 0.707	0.856
Q6	0.24 ± 0.513	0 (0–2)	0.29 ± 0.502	0 (0–2)	0.05 ± 0.465	0.366
Q7	0.94 ± 0.988	1 (0–3)	0.96 ± 0.894	1 (0–3)	0.02 ± 0.735	0.833
Q8	0.16 ± 0.367	0 (0–1)	0.14 ± 0.401	0 (0–2)	−0.02 ± 0.424	0.739

Values are given as mean ± standard deviation or as median (minimum–maximum). ESS: Epworth sleepiness scale, CPAP: continuous positive airway pressure, Q: question. *: *p* < 0.05.

**Table 3:** Baseline (patient- and partner-reported) ESS score in OSA severity groups.

OSA Severity	Number of Patients	Patient-Reported ESS Score		Partner-Reported ESS Score		*p* Value
		Mean	Median	Mean	Median	
Mild	6 (11.8)	12.67 ± 7.50	15.5 (1–19)	12.50 ± 4.68	15 (6–16)	0.786
Moderate	19 (37.3)	10.68 ± 4.84	11 (3–19)	11.53 ± 4.41	11 (5–18)	0.273
Severe	26 (51.0)	10.35 ± 5.18	10.5 (1–19)	11.19 ± 5.53	12 (1–19)	0.253

Values are given as number (%), as mean ± standard deviation or as median (minimum–maximum). ESS: Epworth sleepiness scale, OSA: obstructive sleep apnoea.

## Data Availability

The data presented in this study are available on request from the corresponding author. The data are not publicly available due to privacy related restrictions.
